# *Prevotella timonensis* Bacteria Associated With Vaginal Dysbiosis Enhance Human Immunodeficiency Virus Type 1 Susceptibility Of Vaginal CD4^+^ T Cells

**DOI:** 10.1093/infdis/jiae166

**Published:** 2024-04-04

**Authors:** Nienke H van Teijlingen, Marleen Y van Smoorenburg, Ramin Sarrami-Forooshani, Esther M Zijlstra-Willems, John L van Hamme, Hanneke Borgdorff, Janneke H H M van de Wijgert, Elisabeth van Leeuwen, Joris A M van der Post, Karin Strijbis, Carla M S Ribeiro, Teunis B H Geijtenbeek

**Affiliations:** Department of Experimental Immunology, Amsterdam University Medical Center, University of Amsterdam, Amsterdam, The Netherlands; Amsterdam Institute for Immunology and Infectious Diseases, Amsterdam, The Netherlands; Department of Obstetrics and Gynecology, Amsterdam University Medical Center, University of Amsterdam, Amsterdam, The Netherlands; Department of Experimental Immunology, Amsterdam University Medical Center, University of Amsterdam, Amsterdam, The Netherlands; Amsterdam Institute for Immunology and Infectious Diseases, Amsterdam, The Netherlands; Department of Experimental Immunology, Amsterdam University Medical Center, University of Amsterdam, Amsterdam, The Netherlands; Advanced Therapy Medicinal Product Department, Breast Cancer Research Center, Motamed Cancer Institute, Academic center for Education, Culture and Research, Tehran, Iran; Department of Experimental Immunology, Amsterdam University Medical Center, University of Amsterdam, Amsterdam, The Netherlands; Amsterdam Institute for Immunology and Infectious Diseases, Amsterdam, The Netherlands; Department of Experimental Immunology, Amsterdam University Medical Center, University of Amsterdam, Amsterdam, The Netherlands; Amsterdam Institute for Immunology and Infectious Diseases, Amsterdam, The Netherlands; Amsterdam Institute for Global Health and Development, Amsterdam, The Netherlands; Department of Public Health and Primary Care, Leiden University Medical Center, Leiden, The Netherlands; Julius Center for Health Sciences and Primary Care, University Medical Center Utrecht, Utrecht University, Utrecht, The Netherlands; Department of Obstetrics and Gynecology, Amsterdam University Medical Center, University of Amsterdam, Amsterdam, The Netherlands; Department of Obstetrics and Gynecology, Amsterdam University Medical Center, University of Amsterdam, Amsterdam, The Netherlands; Department of Biomolecular Health Sciences, Division of Infectious Diseases and Immunology, Faculty of Veterinary Medicine, Utrecht University, Utrecht, The Netherlands; Department of Experimental Immunology, Amsterdam University Medical Center, University of Amsterdam, Amsterdam, The Netherlands; Amsterdam Institute for Immunology and Infectious Diseases, Amsterdam, The Netherlands; Department of Experimental Immunology, Amsterdam University Medical Center, University of Amsterdam, Amsterdam, The Netherlands; Amsterdam Institute for Immunology and Infectious Diseases, Amsterdam, The Netherlands

**Keywords:** CD4^+^ T cells, HIV-1 susceptibility, microbiome, vaginal dysbiosis, *Prevotella timonensis*

## Abstract

Dysbiosis of the vaginal microbiome poses a serious risk for sexual human immunodeficiency virus type 1 (HIV-1) transmission. *Prevotella* spp are abundant during vaginal dysbiosis and associated with enhanced HIV-1 susceptibility; however, underlying mechanisms remain unclear. Here, we investigated the direct effect of vaginal bacteria on HIV-1 susceptibility of vaginal CD4^+^ T cells. Notably, pre-exposure to *Prevotella timonensis* enhanced HIV-1 uptake by vaginal T cells, leading to increased viral fusion and enhanced virus production. Pre-exposure to antiretroviral inhibitors abolished *P timonensis*–enhanced infection. Our study shows that the vaginal microbiome directly affects mucosal CD4^+^ T-cell susceptibility, emphasizing importance of vaginal dysbiosis diagnosis and treatment.

## Teaser

Exposure to *Prevotella timonensis*, a bacterium associated with vaginal dysbiosis, increased uptake, fusion, and production of HIV-1 by vaginal CD4^+^ T cells, thereby enhancing HIV-1 susceptibility. Pre-exposure of CD4^+^ T cells to antiretroviral inhibitors abolished *P timonensis*–enhanced HIV-1 infection.

Reducing human immunodeficiency virus type 1 (HIV-1) acquisition among sub-Saharan African adolescent girls and young women remains one of the biggest challenges in the fight against HIV-1 and AIDS [1]. The vast majority of cisgender women living with HIV-1 become infected through heterosexual intercourse. Dysbiosis of the vaginal microbiome greatly increases the risk for HIV-1 acquisition [2–4]. A healthy microbiome is dominated by *Lactobacillus* spp, such as *L crispatus*, whereas in vaginal dysbiosis the microbiome consists of different bacterial species including *Gardnerella vaginalis*, *Fannyhessea vaginae*, *Megasphaera elsdenii*, and *Prevotella* spp [5, 6]. *Prevotella* spp have been associated with increased susceptibility to HIV-1 [2, 4]. This could in part be explained by enhanced influx of activated CD4^+^ T cells, as shown by murine studies with colonization of *Prevotella* spp [4]. However, specific *Prevotella* spp can also directly affect susceptibility of cells, as we have shown recently that *Prevotella timonensis* turns antiviral human vaginal Langerhans cells into HIV-1 reservoirs that transmit HIV-1 to CD4^+^ T cells [7]. *Prevotella timonensis*–exposed Langerhans cells were not productively infected but instead sequestered HIV-1 into specialized vesicles, which resulted in enhanced Langerhans cell–mediated release of infectious virus [7]. Here, we assessed HIV-1 susceptibility of human vaginal CD4^+^ T cells in the presence of various vaginal bacteria.

## MATERIALS AND METHODS

### Study Approval

Human vaginal tissue from women undergoing vaginal surgery for pelvic organ prolapse was collected. In this procedure, excessive vaginal tissue from anterior or posterior vaginal wall was removed. Approval for this study, which included the tissue harvesting procedures, was granted by the Medical Ethics Review Committee of the Amsterdam University Medical Center in The Netherlands (reference number W13_046 # 13.17.0060). All samples were handled in accordance with relevant regulations and guidelines. Clinical and personal information of the participants is not available.

### Vaginal Tissue Preparation and CD4^+^ T-Cell Isolation

Vaginal tissue was freshly processed for each experiment. Surplus stroma was dissected until a thin layer of submucosa remained and tissue was cut into strips of 7 mm. Vaginal tissue strips were incubated overnight at 4°C in complete medium (Iscoves modified Dulbecco's medium of Thermo Fisher Scientific with L-glutamine 100 mM, 10% fetal calf serum, 2500 U/mL penicillin, and 2500 mg/mL streptomycin) supplemented with Dispase II (3 U/mL, Roche Diagnostics). After incubation, the lamina propria was removed from the epithelial layer and epithelial sheets were extensively washed and placed in a Transwell system (Corning, 6.5 mm Transwell 5.0 µm pore polycarbonate membrane inserts) containing complete medium, overnight stimulated with bacteria, and infected with HIV-1. After 3 days, the emigrated fraction of the epithelial layer was collected and stained. As experimental conditions could affect CD4 staining, CD3 expression was used during flow cytometry analysis to assess the CD4^+^ T-cell population in the migratory fraction. This has resulted in a slight underrepresentation of HIV-1 infection percentages among CD4^+^ T cells in our explant model as CD3^+^ cells contain some CD8^+^ T cells.

Peripheral blood mononuclear cells (PBMCs) were isolated from whole blood using Ficoll gradient centrifugation (Axis-shield) and stimulated overnight in complete medium supplemented with 1 µg/mL phytohemagglutinin (PHA, Welcome) to create eligible HIV-1 targets. CD4^+^ T cells were isolated using a CD4^+^ T-cell isolation kit (MACS, Miltenyi Biotec) and were routinely >95% pure. Isolated CD4^+^ T cells were either used freshly or after freeze-thawing and recovery overnight in interleukin 2 (20 U/mL)–supplemented medium. Cells (5 × 10^4^/well) were placed in a 96-well plate (Corning) and stimulated accordingly.

### Vaginal Bacteria, Stimuli, and Inhibitors

*Lactobacillus crispatus* (German Collection of Microorganisms and Cell Cultures GmbH [DSMZ]-20584), *Lactobacillus iners* (DSMZ-13335), *Gardnerella vaginalis* (DSMZ-4944), *Fannyhessea vaginae* (DMSZ-15829), *Megasphaera elsdenii* (DSMZ-20460), *Bacteroides fragilis* (ATCC-25285), *Bacteroides thetaiotaomicron* (DSMZ-2079), *Escherichia coli* (NC0749147), *Prevotella amnii* (DSMZ-23384), *Prevotella bivia* (DSMZ-20514), *Prevotella copri* (or *Segatella copri*; DSMZ-18205), *Prevotella intermedia* (DSMZ-20706), and *Prevotella timonensis* (or *Hoylesella timonensis*; DSMZ-22865) were cultured as recommended by DSMZ. After harvesting bacteria during log-phase growth, bacteria were extensively washed in phosphate-buffered saline and brought to an optical density at 600 nm (OD_600_) of 1. Culture purity and Gram stain were determined. Subsequently, the bacterial suspension was UV-inactivated. Loss of viability was verified by plating UV-inactivated samples. CD4^+^ T cells were stimulated at multiplicity of infection 10. 5 × 10^5^ bacteria were used for stimulation of vaginal explants. In addition, CD4^+^ T cells were stimulated with lipopolysaccharide (LPS) derived from *E coli* (10 ng/mL, Sigma) or *Salmonella typhosa* (10 ng/mL, Sigma), and prior to bacterial stimulation with 10 µg/mL TLR4 blocking antibody (7E3, Hycult). Both vaginal explants and isolated CD4^+^ T cells were stimulated overnight before HIV-1 infection.

### Viruses

HIV-1 SF162 was obtained from Dr Jay Levy. SF162, NL4.3, NL4.3-BaL-BlaM-Vpr, and NL4.3-BaL-eGFP HIV-1 were generated and titrated as described previously [8].

### Data and Statistical Analyses

FACS data analysis was carried out with FlowJo version 10 (TreeStar) software, and statistical analyses were performed using GraphPad Prism version 9.5 software. Two-tailed *t* tests for unpaired observations were performed. Symbols represent independent donors, and bars represent mean ± standard deviation. Significance was set at *P* < .05.

## RESULTS

**Figure 1.**
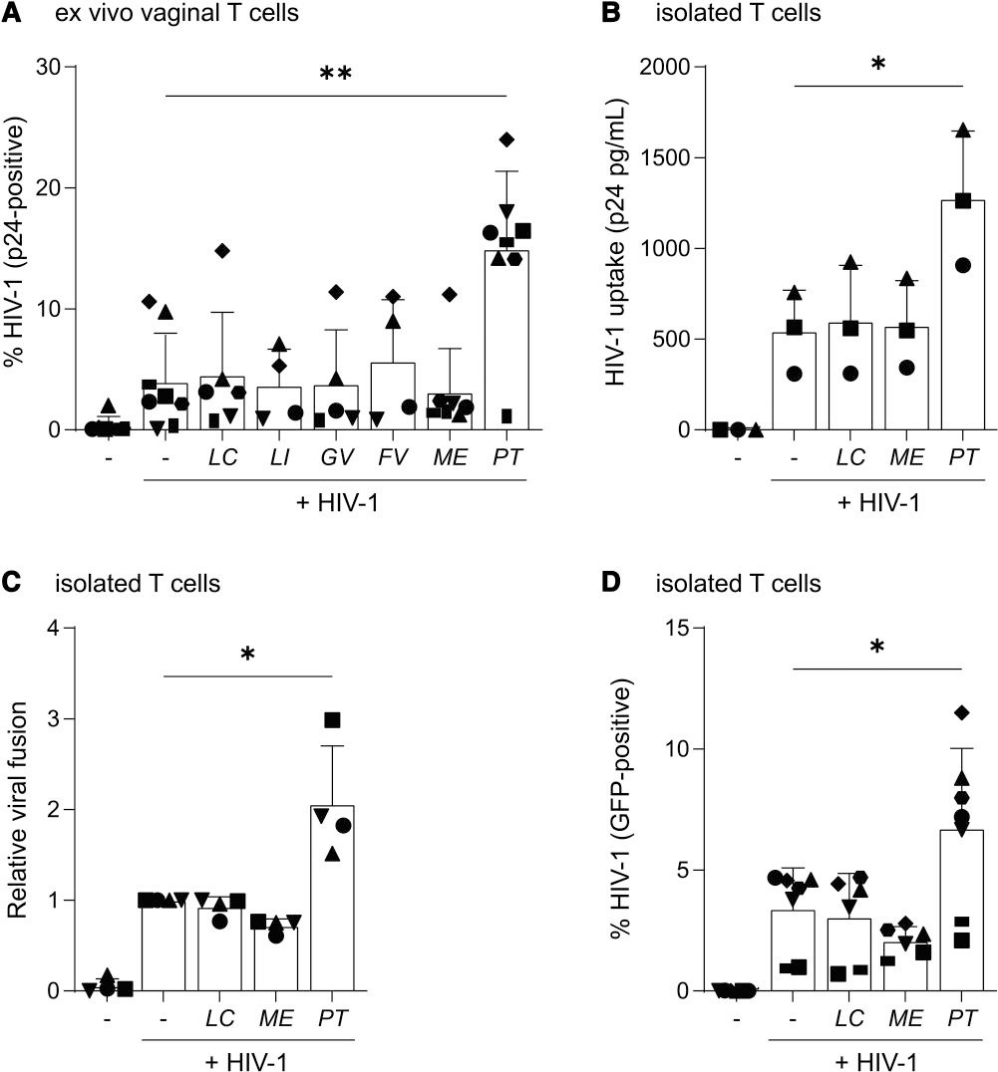
*Prevotella timonensis*–induced human immunodeficiency virus type 1 (HIV-1) uptake, fusion, and viral replication in vaginal CD4^+^ T cells. Vaginal epithelium explants (*A*) or CD4^+^ T cells isolated from phytohemagglutinin-stimulated peripheral blood mononuclear cells (*B–D*) were stimulated overnight by UV-inactivated bacteria (*Lactobacillus crispatus* [LC], *Lactobacillus iners* [LI], *Gardnerella vaginalis* [GV], *Fannyhessea vaginae* [FV], *Megasphaera elsdenii* [ME], and *Prevotella timonensis* [PT], all on multiplicity of infection [MOI] 10) and subsequently exposed to HIV-1 (SF162; MOI 0.1) for 3 d unless stated differently. *A*, HIV-1 infection was measured by flow cytometry after intracellular staining for HIV-1 capsid p24 and depicted here as % p24^+^ cells of CD3^+^ cells of emigrated fraction (explant model, n = 4–8). *B*, HIV-1 uptake in CD4^+^ T cells after 4 h HIV-1 exposure as measured by p24 enzyme-linked immunosorbent assay after trypsin treatment and cell lysis (n = 3). *C*, Pooled data of β-lactamase activity measured by flow cytometry, representing viral fusion upon 4 h infection with NL4.3BaL-BlaM-Vpr (n = 4). *D*, De novo HIV-1 replication, determined by detecting green fluorescent protein, after HIV-1 NL4.3eGFP-BaL infection (n = 6–7). Symbols represent independent donors, bars represent mean ± standard deviation. *P < .05, **P < .01, 2-tailed *t* test.

Primary human vaginal explants were exposed to selected UV-killed bacteria and subsequently infected by HIV-1 as previously described [7]. After 3 days, emigrated cells from tissue were isolated and analyzed for expression of T-cell marker CD3 and HIV-1 capsid protein (p24). About 5% of emigrated T cells stained positive for p24. Similar numbers of p24^+^ T cells were detected after exposure of vaginal explants to *L crispatus*, *L iners*, *G vaginalis*, *F vaginae*, and *M elsdenii* (Figure 1*A*). Notably, exposure to *P timonensis* significantly increased the number of HIV-1–positive T cells (Figure 1*A*). Next, CD4^+^ T cells isolated from PHA-activated PBMCs were infected with HIV-1 after exposure to different vaginal bacteria. Interestingly, *P timonensis* increased HIV-1 susceptibility of CD4^+^ T cells whereas the other bacteria did not affect the number of CD4^+^ T cells staining positive for HIV-1 p24 capsid protein (Supplementary Figure 1). These data strongly suggest that *P timonensis* acts directly on CD4^+^ T cells and increases HIV-1 infection.

Next, we investigated the impact of *P timonensis* on HIV-1 uptake, which was examined by determining capsid p24 levels by enzyme-linked immunosorbent assay in cell lysates of CD4^+^ T cells that were exposed to bacteria prior to HIV-1 infection. *Prevotella timonensis* significantly increased HIV-1 uptake in CD4^+^ T cells compared to other tested bacteria (Figure 1*B*). Neither related *Prevotella* spp, nor *Bacteroides* spp, induced HIV-1 uptake in CD4^+^ T cells (Supplementary Figure 2). In addition, *P timonensis*–enhanced uptake was independent of bacterial LPS and TLR4 signaling (Supplementary Figure 2). Furthermore, blocking CD4 did not abrogate the *P timonensis*–induced HIV-1 uptake even though HIV-1 uptake was decreased in both untreated and *P timonensis*–treated T cells (Supplementary Figure 3). While *P timonensis* increased CD4 expression, it did not induce expression of co-receptors CCR5 and CXCR4 nor upregulation of T-cell activation markers CD25 and CD69 (Supplementary Figures 4 and 5). In addition, *P timonensis* enhanced uptake of X4-tropic virus NL4.3 similar to uptake of R5-tropic SF162 (Supplementary Figure 6). These data suggest *P timonensis* enhances HIV-1 uptake, which is independent of CD4, co-receptor usage, and TLR4 signaling.

Next, we investigated the impact of *P timonensis* on the consequent stages of the HIV-1 replication cycle. To assess HIV-1 fusion to the membrane of CD4^+^ T cells, we employed a HIV-1-BlaM-vpr-based fusion assay [7]. Additionally, we investigated de novo virus production using a pseudotyped green fluorescent protein–reporter virus [7]. Notably, *P timonensis* strongly enhanced HIV-1 fusion to CD4^+^ T cells (Figure 1*C*, Supplementary Figure 1) accompanied by increased de novo viral synthesis (Figure 1*D*). Taken together, our data suggest that *P timonensis* enhances HIV-1 uptake, fusion, and translation, resulting in increased productive HIV-1 infection of CD4^+^ T cells.

**Figure 2.**
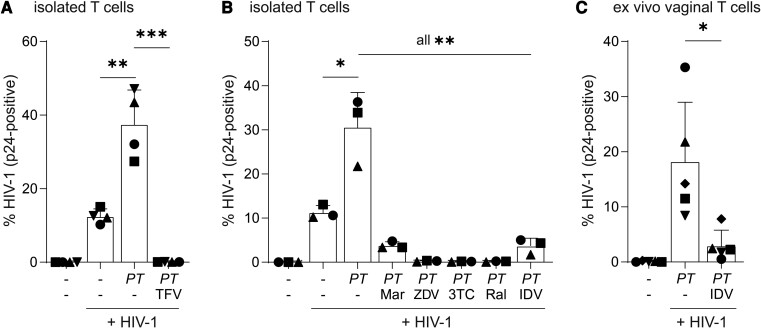
Antiretroviral drugs used in pre-exposure prophylaxis (PrEP) and combination antiretroviral therapy (cART) block *Prevotella timonensis*–enhanced human immunodeficiency virus type 1 (HIV-1) infection in vaginal CD4^+^ T cells. *A–C*, CD4^+^ T cells isolated from phytohemagglutinin (PHA)–stimulated peripheral blood mononuclear cells (PBMCs) (*A* and *B*) or vaginal epithelium explants (*C*) were stimulated overnight by UV-inactivated bacteria (*Lactobacillus crispatus* [LC], *Lactobacillus iners* [LI], *Gardnerella vaginalis* [GV], *Fannyhessea vaginae* [FV], *Megasphaera elsdenii* [ME], and *Prevotella timonensis* [PT], all on multiplicity of infection [MOI] 10) and subsequently exposed to HIV-1 (SF162; MOI 0.1) for 3 d. HIV-1 infection was measured by flow cytometry after intracellular staining for HIV-1 capsid p24 and depicted here as % p24^+^ cells of total cells (CD4^+^ T cells isolated from PHA-stimulated PBMCs (*A* and *B*) or CD3^+^ cells of emigrated fraction (explant model, *C*). HIV-1 infection in the presence or absence of the PrEP drug tenofovir (TFV, reverse-transcriptase inhibitor, 50 µM, *A*, n = 4) or replication inhibitors used in cART (*B*, n = 3; *C*, n = 5); maraviroc (Mar, CCR5 blockage, 30 µM); reverse transcriptase inhibitors zidovudine (ZDV, 20 µM) and lamivudine (3TC, 50 µM); raltegravir (Ral, integrase inhibitor, 100 nM); and indinavir (IDV, protease inhibitor, 5 µM). Symbols represent independent donors, bars represent mean ± standard deviation. *P < .05, **P < .01, ***P < .001, 2-tailed *t* test.

Consequently, we investigated whether the drugs used for pre-exposure prophylaxis (PrEP) or in combination antiretroviral treatment (cART) influenced *P timonensis-*enhanced HIV-1 susceptibility of CD4^+^ T cells. Interestingly, the PrEP drug tenofovir abolished *P timonensis*–increased infection of CD4^+^ T cells (Figure 2*A*). Moreover, viral inhibitors used in cART showed partial (maraviroc and indinavir) or complete (zidovudine, lamivudine, and raltegravir) block of *P timonensis*–enhanced HIV-1 infection of CD4^+^ T cells (Figure 2*B*). Furthermore, indinavir showed significant reduction of *P timonensis–*induced HIV-1 infection of CD4^+^ T cells migrating from vaginal explants (Figure 2*C*), demonstrating therapeutic potential for antiretrovirals in prevention of HIV-1 transmission during vaginal dysbiosis.

## DISCUSSION

Previous studies have suggested that *Prevotella* spp affect HIV-1 susceptibility by increasing inflammation and influx of T cells [4, 9]. Our data strongly suggest that *P timonensis* has a direct effect on T-cell infection and thereby increases vaginal HIV-1 susceptibility. *Prevotella* spp increase HIV-1 uptake by ex vivo vaginal T cells. Importantly, fusion and productive infection were also increased by *P timonensis*, whereas none of the other tested bacteria affected HIV-1 susceptibility. *Prevotella timonensis* is one of the most abundant species present during vaginal dysbiosis [10, 11]. Besides colonizing the vagina during dysbiosis, *P timonensis* has also been detected in oral and anal swabs [10]. In addition, *Prevotella* are the most abundant bacterial species present in the penile microbiome, and penile colonization by *Prevotella* spp, including *P timonensis*, is significantly associated with enhanced risk of seroconversion [12]. Our data indicate that *P timonensis* directly increases HIV-1 uptake and productive infection. Altogether, this suggests *P timonensis* could influence HIV-1 susceptibility and transmission at multiple anatomic sites.

We observed donor variability with regard to HIV-1 uptake, which could be due to differences in age and hormonal levels that can affect immune cell numbers, cellular activation state, and therefore HIV-1 susceptibility. However, as the same donor tissue was exposed to different vaginal bacteria, our data suggest that irrespective of donor or clinical characteristics, *P timonensis* enhanced the number of p24^+^ vaginal as well as blood-derived CD4^+^ T cells. Taken together, this suggests that *P timonensis*–enhanced susceptibility is independent of sex, hormonal state, and age.

We observed that *P timonensis* enhanced productive infection of CD4^+^ T cells but not infection of immature Langerhans cells [7], even though *P timonensis* stimulated HIV-1 uptake in both T cells and Langerhans cells. As vaginal CD4^+^ T cells are permissive whereas vaginal Langerhans cells are naturally restrictive to HIV-1 [7, 13, 14], our data suggest that *P timonensis* increases HIV-1 uptake, which results in enhanced virus infection only in HIV-1 permissive cells. Interestingly, antiretroviral inhibitors blocked enhanced infection of vaginal and PBMC-isolated CD4^+^ T cells after *P timonensis* exposure, underscoring their importance in preventing HIV-1 transmission.

Dysbiosis of the vaginal microbiome greatly enhances susceptibility to HIV-1 acquisition in young women [2–4]. Vaginal dysbiosis alters the local immune environment and enhances inflammation in the vaginal mucosa, resulting in a disrupted barrier function as well as enhanced influx of activated CD4^+^ T cells, both enhancing susceptibility to HIV-1 infection [4, 9]. In women of reproductive age, the vagina was estimated to contain 10^10^ to 10^11^ bacteria [15]. Furthermore, vaginal dysbiosis leads to a loss of *Lactobacillus* spp and an increase of 100- to 1000-fold in concentration of dysbiosis-associated bacteria, such as *Prevotella* spp [15], emphasizing that mucosal immune cells are exposed to high numbers of bacteria during vaginal dysbiosis. Here we have shown that *P timonensis* present in vaginal dysbiosis directly affects HIV-1 susceptibility, by enhancing HIV-1 infection of CD4^+^ T cells. Other members of vaginal bacterial communities not tested in this study might have similar enhancing effects on vaginal HIV-1 susceptibility. Follow-up studies using a broader range of vaginal bacteria will help reveal whether additional species are also able to enhance HIV-1 susceptibility.

Taken together, our data show that multiple target cells across the vaginal tissue are sensitive to *P timonensis* exposure, suggesting a broad-acting mechanism. It will be important to identify the molecular mechanism induced by *P timonensis* triggering enhanced viral uptake. This will help in understanding the general underlying bacterial mechanism eliciting this effect. Treatment of symptomatic vaginal dysbiosis, and even specifically screening for *P timonensis*, could be important to understand HIV-1 acquisition risk. Moreover, *Prevotella* spp are also present in asymptomatic vaginal dysbiosis, supporting the importance of also treating asymptomatic vaginal dysbiosis in populations at risk for HIV-1 acquisition. Ultimately, our work underscores the need for better identification, prevention, and treatment of women with vaginal dysbiosis, particularly those carrying *P timonensis*.

## Supplementary Data

Supplementary materials are available at *The Journal of Infectious Diseases* online (http://jid.oxfordjournals.org/). Supplementary materials consist of data provided by the author that are published to benefit the reader. The posted materials are not copyedited. The contents of all supplementary data are the sole responsibility of the authors. Questions or messages regarding errors should be addressed to the author.

## Supplementary Material

jiae166_Supplementary_Data

## Notes

***Data availability.*** Detailed data are available from the corresponding author upon request.

***Financial support*.** This work was supported by the Amsterdam University Medical Center PhD Scholarship (to N. H. v. T.); Dutch Research Council (NWO-ZonMW) TOP grant 91218017; and European Research Council Advanced Grant 670424 (to T. B. H. G.).
