# Supplementary material for: Prevotella timonensis Bacteria Associated With Vaginal Dysbiosis Enhance Human Immunodeficiency Virus Type 1 Susceptibility Of Vaginal CD4+ T Cells
Source: J Infect Dis. 2024 Apr 4;230(1):e43–7. doi: 10.1093/infdis/jiae166 (PMC11272099; doi:10.1093/infdis/jiae166)
Supplement: jiae166_Supplementary_Data [file jiae166_supplementary_data.zip › Supplementary_Figure_1.docx]

**
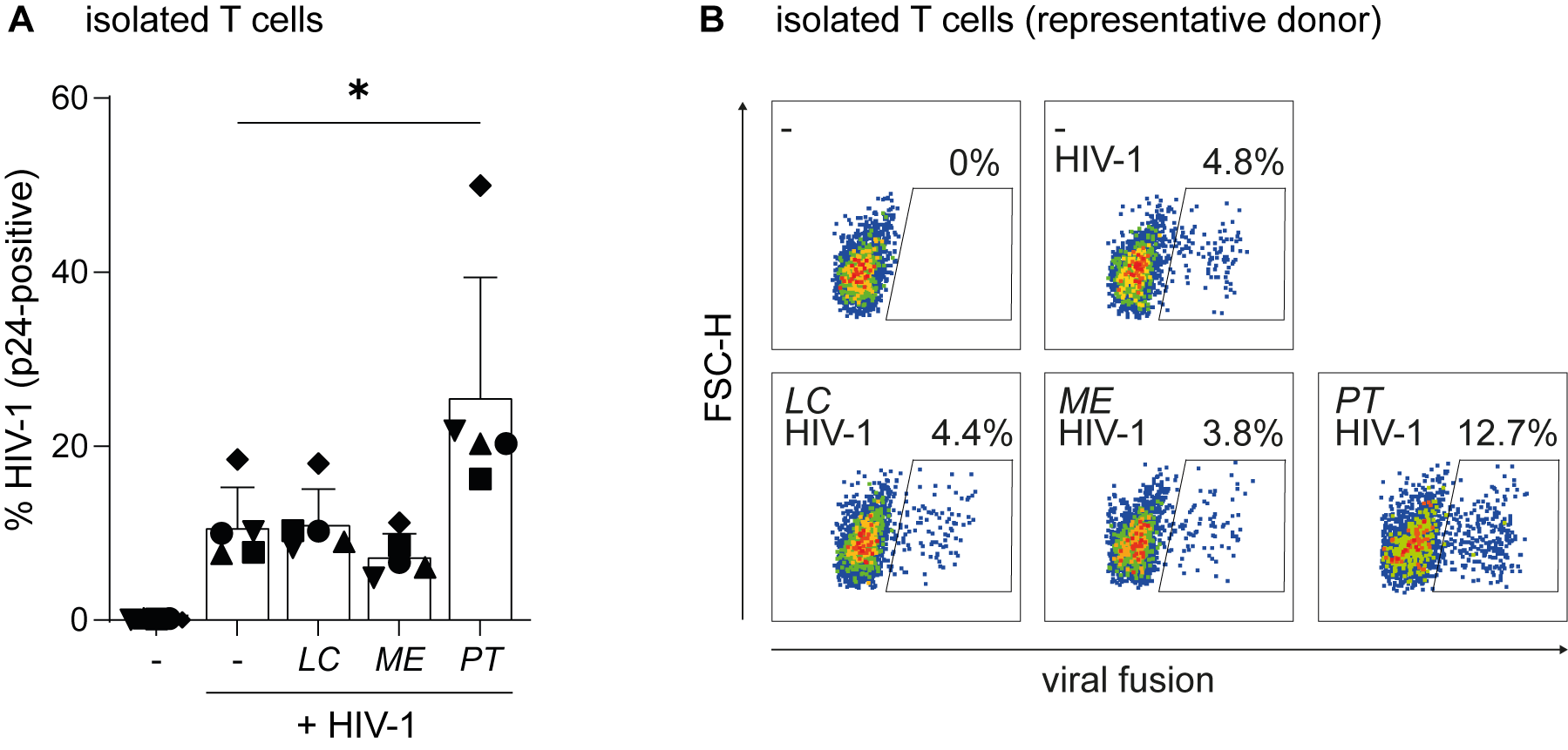
**

**Supplementary Figure 1. *P. timonensis*-induced HIV-1 uptake and fusion in CD4^+^ T cells.**

CD4^+^ T cells isolated from PHA-stimulated PBMCs were stimulated O/N by UV-inactivated bacteria (*Lactobacillus crispatus* (LC), *Megasphaera elsdenii* (ME), and *Prevotella timonensis* (PT) all on MOI 10) and subsequently exposed to HIV-1 (SF162; MOI 0.1). **A.** HIV-1 infection was measured after 3 days by flow cytometry after intracellular staining for HIV-1 capsid p24 and depicted here as % p24^+^ cells (N=5). **B.** Representative donor depicting ß-lactamase activity measured by flow cytometry, representing viral fusion upon 4h infection with NL4.3BaL-BlaM-Vpr. Symbols represent independent donors, bars represent mean ± SD. **P* < 0.05, two-tailed *t-*test.
