# Supplementary material for: Prevotella timonensis Bacteria Associated With Vaginal Dysbiosis Enhance Human Immunodeficiency Virus Type 1 Susceptibility Of Vaginal CD4+ T Cells
Source: J Infect Dis. 2024 Apr 4;230(1):e43–7. doi: 10.1093/infdis/jiae166 (PMC11272099; doi:10.1093/infdis/jiae166)
Supplement: jiae166_Supplementary_Data [file jiae166_supplementary_data.zip › Supplementary_Figure_2.docx]

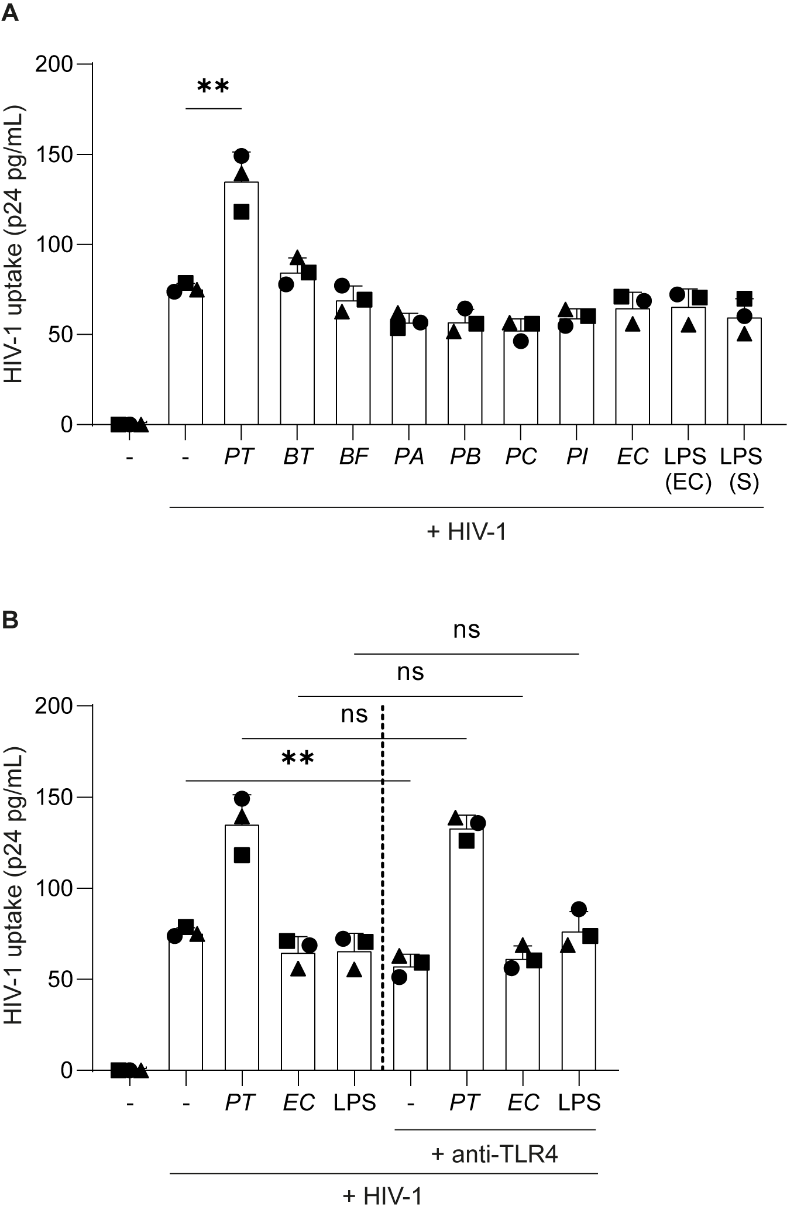


**Supplementary Figure 2. *P. timonensis*-enhanced HIV-1 uptake is independent of bacterial LPS and TLR4 signalling.**

Activated CD4^+^ T cells were stimulated O/N by UV-inactivated bacteria (MOI 10) and subsequently exposed to HIV-1 (SF162; MOI 0.1). After 4h exposure, cells were lysed and HIV-1 uptake was measured by p24 ELISA (N=3). **A.** CD4^+^ T cells were stimulated O/N with *Bacteroides fragilis* (BF), *Bacteroides thetaiotaomicron* (BT), *Escherichia coli* (EC), *Prevotella amnii* (PA), *Prevotella bivia* (PB), *Prevotella copri* (PC), *Prevotella intermedia* (PI), *P. timonensis* (PT), LPS derived from *E. coli* (10ng/ml) or LPS derived from *Salmonella typhosa* (10ng/ml) (N=3). **B.** CD4^+^ T cells were pre-treated with blocking TLR4 antibody (10µg/ml) 30 minutes prior to O/N stimulation with PT, EC or LPS derived from *E. coli* (10ng/ml) (N=3). Symbols represent independent donors, bars represent mean ± SD. Ns, not significant, ***P* < 0.01, two-tailed *t-*test.
