# Supplementary material for: Prevotella timonensis Bacteria Associated With Vaginal Dysbiosis Enhance Human Immunodeficiency Virus Type 1 Susceptibility Of Vaginal CD4+ T Cells
Source: J Infect Dis. 2024 Apr 4;230(1):e43–7. doi: 10.1093/infdis/jiae166 (PMC11272099; doi:10.1093/infdis/jiae166)
Supplement: jiae166_Supplementary_Data [file jiae166_supplementary_data.zip › Supplementary_Figure_3.docx]

**
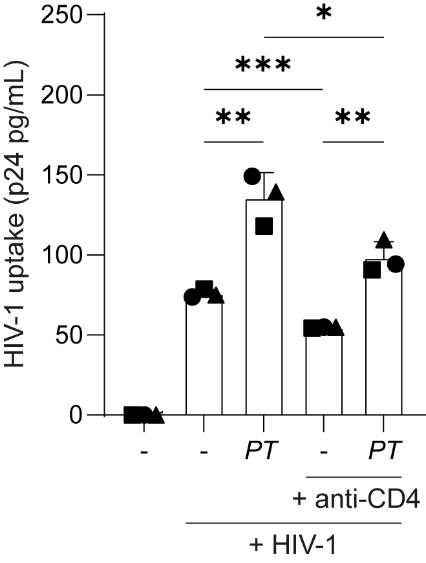
**

**Supplementary Figure 3. Blocking CD4 did not abrogate *P. timonensis*-induced HIV-1 uptake in CD4^+^ T cells.**

Activated CD4^+^ T cells were stimulated O/N by UV-inactivated *P. timonensis* (PT, MOI 10), incubated with a CD4 blocking antibody (20µg/ml) for 1h and subsequently exposed to HIV-1 (SF162; MOI 0.1). After 4h exposure, cells were lysed and HIV-1 uptake was measured by p24 ELISA (N=3). Symbols represent independent donors, bars represent mean ± SD.**P* < 0.05, ***P* < 0.01, ****P* < 0.001, two-tailed *t-*test.
