# Supplementary material for: Prevotella timonensis Bacteria Associated With Vaginal Dysbiosis Enhance Human Immunodeficiency Virus Type 1 Susceptibility Of Vaginal CD4+ T Cells
Source: J Infect Dis. 2024 Apr 4;230(1):e43–7. doi: 10.1093/infdis/jiae166 (PMC11272099; doi:10.1093/infdis/jiae166)
Supplement: jiae166_Supplementary_Data [file jiae166_supplementary_data.zip › Supplementary_Figure_6.docx]

**
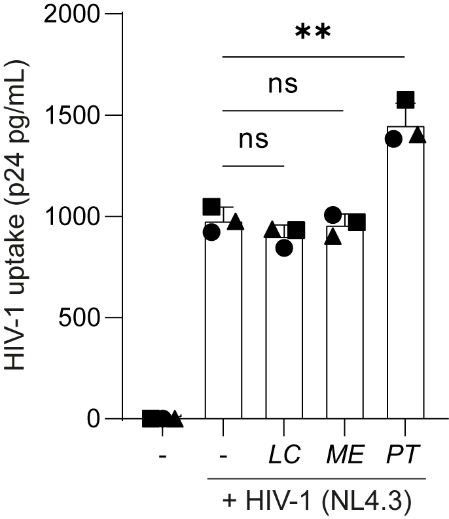
**

**Supplementary Figure 6. *P. timonensis* enhanced X4-tropic HIV-1 uptake in CD4^+^ T cells.**

Activated CD4^+^ T cells were stimulated O/N by UV-inactivated bacteria (*L. crispatus* (LC), *M. elsdenii* (ME), and *P. timonensis* (PT) all on MOI 10) and subsequently exposed to X4-tropic HIV-1 (NL4.3; MOI 0.1). After 4h exposure, cells were lysed and HIV-1 uptake was measured by p24 ELISA (N=3). Symbols represent independent donors, bars represent mean ± SD. Ns, not significant, ***P* < 0.01, two-tailed *t-*test.
