# Supplementary material for: Prevotella timonensis Bacteria Associated With Vaginal Dysbiosis Enhance Human Immunodeficiency Virus Type 1 Susceptibility Of Vaginal CD4+ T Cells
Source: J Infect Dis. 2024 Apr 4;230(1):e43–7. doi: 10.1093/infdis/jiae166 (PMC11272099; doi:10.1093/infdis/jiae166)
Supplement: jiae166_Supplementary_Data [file jiae166_supplementary_data.zip › Supplementary_Materials_and_Methods.docx]

**Supplementary Materials and Methods**

*HIV-1 infection experiments*

5x10^4^ purified CD4^+^ T cells were infected with MOI 0.1 and explants were exposed to volumes equivalent to volumes used for 5x10^4^ purified CD4^+^ T cells, after overnight stimulation with bacteria or medium. HIV-1 p24 content and HIV-1-eGFP expression were assessed at day 3 of HIV-1 exposure by flow cytometry. Samples were fixed using 4% (w/v) paraformaldehyde, permeabilised using PBS supplemented with 0.5% saponin (Sigma) and 0.5% BSA (Sigma) for intracellular staining, stained for CD3-PercP (SP34-2, BD Pharmingen) and p24-PE (KC57, Beckman Coulter) and analysed using FACSCanto II flow cytometers (BD Biosciences). HIV-1 acquisition was defined as % p24^+^ CD3^+^ cells.

*HIV-1 uptake assay*

After overnight stimulation with medium or bacteria, CD4^+^ T cells were incubated for 4 hours with SF162 (MOI 0.1). Samples were then treated with Trypsin-EDTA (0.05%, Invitrogen) to remove membrane-bound HIV-1 virions of input-virus, washed extensively and lysed. HIV-1 uptake was quantified by determining p24-antigen levels (pg/mL) of these lysates by ELISA according to manufacturer’s instructions (ZeptoMetrix).

*HIV-1 fusion assay*

A BlaM-Vpr-based assay was used to quantify fusion of HIV-1 to the CD4^+^ T cell membrane as previously described [1]. In short; CD4^+^ T cells were stimulated overnight with bacteria followed by infection with NL4.3BaL-BlaM-Vpr for 4 hours. Next, CD4^+^ T cells were loaded with substrate CCF2/AM (Life technologies) in serum-free medium for 1 hour and BLaM-reaction was allowed to develop overnight at 22 °C in complete medium supplemented with probenecid (Sigma Pharmaceuticals). The shift from green (520nm) to blue (450nm) fluorescence, reflecting the release of BlaM-Vpr into the CD4^+^ T cell cytoplasm after fusion, was assessed using flow cytometry (LSRFortessa, BD Biosciences).

*HIV-1 replication inhibitors*

CD4^+^ T cells were 1 hour prior to HIV-1 infection incubated with tenofovir (TFV, reverse-transcriptase (RT) inhibitor, 50 µM); maraviroc (Mar, CCR5 blockage, 30 µM); reverse-transcriptase inhibitors zidovudine (ZDV, 20 µM) and lamivudine (3TC, 50 µM); raltegravir (Ral, integrase inhibitor, 100 nM); indinavir (IDV, protease inhibitor, 5 µM) or with 20µg/ml CD4 blocking antibody (RPA-T4, Biolegend, 300502). Vaginal explants were exposed to IDV (5 µM) 1 hour prior to HIV-1 infection.

*T cell activation analysis*

Activated CD4^+^ T cells were stimulated overnight with vaginal bacteria and surface staining was performed in TSM (TSM (20mM Tris, 150mM NaCl, 1mM CaCl_2_, 2mM MgCl_2_) at pH 7.4) supplemented with 0.5% BSA (Sigma) using the (anti-human) antibodies CD3-PercP (SP34-2, BD Pharmingen, 552851), CD4-APC (RPA-T4, BD Pharmingen, 561840), CD25-PE (BC96, BioLegend, 302606), CD69-FITC (FN50, BioLegend, 310904) and analysed using FACSCanto II flow cytometers (BD Biosciences). Expression of each marker was depicted by geometric mean of the fluorescence intensity (MFI).

*RNA isolation and quantitative real-time PCR*

Cells exposed overnight to vaginal bacteria were lysed and mRNA was isolated with the mRNA Catcher™ PLUS Purification Kit (ThermoFisher). Subsequently, mRNA was reversely transcribed to cDNA with a reverse-transcriptase kit (Promega). Quantitative real-time PCR was performed using SYBR Green (ThermoFisher) with an ABI 7500 Fast Real-time PCR System (Applied Biosystems). Expression of genes of interested were calculated using Ct values and normalised to Ct values of household gene GAPDH according to the formula N_t_ = 2^Ct(GAPDH)-Ct(target)^. The Primer Express™ Software v2.0 (Applied Biosystems) was used for designing primers. The following primers were used:
CCR5: Fw_TACCTGCTCAACCTGGCCAT; Rv_ TTCCAAAGTCCCACTGGGC;

CXCR4: Fw_ GCATGACGGACAAGTACAGGC; Rv_ GACAAAGAGGAGGTCGGCC;

GAPDH: Fw_CCATGTTCGTCATGGGTGTG; Rv_ GGTGCTAAGCAGTTGGTGGTG.

**References**

1. Ribeiro CM, Sarrami-Forooshani R, Setiawan LC, et al. Receptor usage dictates HIV-1 restriction by human TRIM5alpha in dendritic cell subsets. Nature **2016**; 540:448-52.
